# Epidemiology of mastocytosis: a population-based study (Sweden)

**DOI:** 10.2340/1651-226X.2024.31406

**Published:** 2024-02-21

**Authors:** Anna Bergström, Hans Hägglund, Anders Berglund, Gunnar Nilsson, Mats Lambe

**Affiliations:** a Department of Medical Sciences, Dermatology and Venereology, Uppsala University, Uppsala Akademiska Hospital, Uppsala, Sweden; b Department of Medical Sciences, Hematology, Uppsala University, Uppsala, Sweden; c Epistat, Uppsala, Sweden; d Department of Medicine, Division of Immunology and Allergy, Karolinska Institutet, Karolinska University Hospital, Stockholm; e Department of Medical Sciences, Hematology, Uppsala University, Uppsala, Sweden; f Department of Medical Epidemiology and Biostatistics, Karolinska Institutet, Stockholm, Sweden; g Regional Cancer Center, Central Sweden, Uppsala, Sweden

**Keywords:** Mastocytosis, epidemiology, comorbidity, population-based, register, Sweden

## Abstract

**Background:**

Mastocytosis is a disease characterized by accumulation of aberrant mast cells and mediator-related symptoms and is divided into systemic mastocytosis (SM) and cutaneous mastocytosis (CM). The epidemiology of mastocytosis remains incompletely understood.

**Objective:**

To estimate the incidence, prevalence, overall survival (OS) and burden of comorbidities in adult mastocytosis patients identified in Swedish population-based registries.

**Methods:**

Individuals (≥ 20 years of age) with a mastocytosis diagnosis in the National Patient Register (NPR) and/or the Swedish Cancer Register (SCR) between 2001 and 2018, were identified. In a matched cohort design, for each case five randomly selected mastocytosis-free comparators matched on age, sex, and county of residence were chosen from the Population Register. The Kaplan-Meier method was used to compare OS between individuals with mastocytosis and comparators. Information on concomitant disease at baseline was assessed by use of the Charlson Comorbidity Index (CCI).

**Results:**

We identified 2,040 adults with a mastocytosis diagnosis yielding an annual incidence of 1.56 per 100,000 (95% CI 1.29–1.87) and a prevalence of 23.9 per 100,000 (95% CI 22.8–25.0). The comorbidity burden was higher, and the OS lower, in patients with mastocytosis compared to comparators.

**Interpretation:**

We found a higher incidence and prevalence of mastocytosis compared to assessments in other settings and confirmed that the prognosis generally is favorable. Of special note was evidence of a higher comorbidity burden in mastocytosis patients compared to the background population.

**Limitations:**

Underreporting and inconsistencies in the use of diagnostic codes.

## Introduction

Mastocytosis is a condition characterized by the focal or generalized proliferation and accumulation of abnormal mast cells (MCs) in different organs and mediator-related symptoms, such as urticaria and flushing [1, 2]. The disease entity includes a heterogeneous group of conditions divided into two major categories: cutaneous mastocytosis (CM) and systemic mastocytosis (SM) [1]. With an improved understanding over time, the WHO diagnostic criteria and classifications for mastocytosis have been refined [3]. In a 2016 update [3], the disease is divided into prognostically different variants of SM, CM and MC sarcoma (MCS). The further classification of SM is based on disease-specific features, separating SM into indolent SM (ISM), smoldering SM (SSM), aggressive SM (ASM), SM with associated hematopoietic neoplasm (SM-AHN) and MC leukemia (MCL). In the most recent update, bone marrow mastocytosis was added as a separate subtype, characterized by the absence of skin lesions [4]. The classification of mastocytosis is of importance since the prognosis in CM is favorable, whereas the prognosis in SM is variable, ranging from indolent, with normal life expectancy, to variants with rapidly deteriorating outcome. CM is categorized into three different major groups: (I) urticaria pigmentosa (UP), also called maculopapular cutaneous mastocytosis (MPCM), (II) diffuse cutaneous mastocytosis (DCM) and (III) cutaneous mastocytoma, where DCM and cutaneous mastocytoma primarily affects children [5]. However, true CM without signs of systemic infiltration – following careful examination – is uncommon in adults [5–7].

The epidemiology of mastocytosis remains incompletely understood. A Dutch study based on 48 patients reported a prevalence of 13 per 100,000 of the most common type of SM (ISM and SSM) [8]. The first population-based study on SM and its subtypes included 548 patients, age 15 years and older, and reported an annual incidence rate and prevalence of 0.89 and 9.59 per 100,000, respectively [9]. In a large Danish study, including 1436 individuals of all ages, an incidence rate of 1.1 cases per 100,000 person years were reported [10]. A recent publication [11] found a prevalence of 10.2 and 17.2 per 100,000 of SM in the adult population in an Italian region and an Italian province, respectively. The mean annual incidence in the Italian province was 1.09 per 100,000. A 15-year retrospective study [12] of adult SM patients diagnosed at the Multidisciplinary Mastocytosis Center in Stockholm, estimated a prevalence of 10.6 per 100,000 inhabitants and a mean annual incidence of 0.77 per 100,000.

Patients with SM are at an increased risk of anaphylaxis [13] and altered bone metabolism, including osteoporosis [14]. Furthermore, there is evidence of an increased risk of melanoma [15, 16]. There have also been reports of an increased risk of solid cancers, venous thromboembolism, stroke [16] and other cardiovascular events [17]. Comorbidities with evidence of a higher baseline prevalence include metastatic cancers, chronic pulmonary diseases and connective tissue diseases [10].

The aim of this study was two-fold. Firstly, to estimate the incidence, prevalence and overall survival (OS) in adult patients identified with a record of a mastocytosis diagnosis in Swedish population-based registers. Secondly, to compare sociodemographic characteristics and the comorbidity burden between individuals with mastocytosis and the background population.

## Methods

### Study design and study population

A dataset was generated by individual level record linkages between Swedish registers with national coverage including the National Patient Register (NPR), the Swedish Cancer Register (SCR) and the Cause of Death Register (CDR). Additional information was retrieved from the Population Register and the longitudinal database on socioeconomic factors (LISA). Record linkages were made possible by use of the unique personal identity number assigned to all residents in Sweden. Hence, the final study population encompassed unique individuals, based on the patient´s first recorded diagnosis.

By use of data in the in- and out-patient Patient Register and the Cancer Register, we identified patients with a record of a primary or secondary diagnosis of mastocytosis in specialist care settings between 2001 and 2018. The NPR includes data based on the International Classification of Diseases (ICD-10). The SCR contains detailed information coded according to the Systematized Nomenclature of Medical-Clinical Terms (SNOMED) (Supplementary, appendix).

We classified SNOMED codes 97403, 97413 and 97423 and ICD-10 codes C943, C962 and 202G as being advanced and SNOMED 97401, 97411 and ICD-codes Q822A/B/C/D and 238F as benign. ICD-codes Q822, Q822X and D470 includes cases not clearly benign or advanced, and hence named ‘mixed’. This classification is used in all analyses regarding subtypes.

Five randomly selected comparators matched on sex, age and county of residence representing the background population were obtained from the Population Register. For both cases and comparators, information on socioeconomic factors and comorbidity burden was obtained from the LISA database and the NPR, respectively.

The LISA database includes continuously updated information on educational level, income, marital status and household size. Education was collapsed into three groups based on the total number of years of schooling: low ≤9 years, middle 10–12 years and high ≥ 13 years, corresponding to mandatory school, high school, and post-high school (college and university).

The comorbidity burden was estimated based on data in the Cancer Register and Hospital inpatient admissions 10 years prior to mastocytosis diagnosis by use of the Charlson Comorbidity Index (CCI) [18]. Based on 17 groups of diseases, the CCI is a commonly used index where each condition is assigned a weight that are summed to obtain an overall score [19]. In this study, the combined CCI score was categorized as 0, 1 and 2+.

Date and cause of death was retrieved from the CDR. Patients were followed until death or at the end of follow-up 31 December 2019, whichever came first.

### Statistical methods

Crude overall incidence per 100,000 person years were estimated. Limited-duration prevalence is the number of people alive on a certain day who were diagnosed with the disease during a specific number of years. Based on study duration, an 18-year limited-duration prevalence was calculated among those still alive on 31 December 2019. Sociodemographic factors and comorbidity burden were compared between cases and comparators. OS was defined from date of diagnosis, death, or end of follow-up. The Kaplan-Meier method was used to compare OS between the cases and individuals free of mastocytosis. In an analysis restricted to cases, OS was compared between benign, mixed and advanced subtypes. Also, each subtype was compared to their sex, age and county of residence matched comparators. All tests were two-sided, and 5% level was considered statistically significant. Statistical analyses were performed using R version 4.1.2.

### Ethics

The study was approved by the Regional Ethical Review board, Uppsala, Sweden (2018/245).

## Results

We identified 4,445 individuals of all ages with at least one record of a mastocytosis diagnosis between 2001 and 2018. The majority of patients had only one registered record, for those with multiple records, the first date of diagnosis was used. Seven individuals lacked a record in the Population Register and were excluded. In this way, the dataset encompassed 4,438 cases and 22,153 comparators of all ages (Supplementary, Table 1). For the purpose of this study, the final study population included 2,040 cases and 10,193 comparators of age 20 years or older. The mean age at diagnosis was 50.6 years, with the majority diagnosed in age-group 40–49 years. There was a clear female predominance (59.4%) (Table 1). Median time of follow-up was 106.1 months (IQR 61.7–163.3).

**Table 1:** Demographic characteristics in 2040 individuals with mastocytosis, age ≥ 20 years at diagnosis.

All subjects	*n*	%
**Sex**		
Male	829	40.6
Female	1211	59.4
**Age at diagnosis**		
Median, IQR	50, 37-64	
Mean, SD	50.6, 17.4	
20–29	284	13.9
30–39	328	16.1
40–49	388	19.0
50–59	365	17.9
60–69	345	16.9
70–79	227	11.1
80–89	91	4.5
90+	12	0.6

### Incidence and prevalence

In individuals aged 20 years or older, the prevalence was 23.9 per 100,000 (95% CI 22.8–25.0) and the cumulative incidence 28.2 (95% CI 27.0–29.5). The mean annual incidence was 1.56 per 100,000 (95% CI 1.29–1.87). The annual incidence varied with evidence of a slightly higher crude incidence rate during the most recent years (2007–2018), compared to the early study period (2001–2006) (Table 2).

**Table 2:** Incidence and prevalence of mastocytosis.

Year	Annual incidence (95% CI)	Prevalence (95% CI)
2001–2018 (full period)	1.56 (1.29–1.87)	23.9 (22.8–25.0)
2001–2006	1.36 (1.11–1.66)	
2007–2018	1.66 (1.39–1.97)	

### Number of patients and subtypes by register source

Of the 2,040 patients aged 20 years or older at diagnosis, 1,912 were identified in the NPR outpatient register, 330 in the NPR inpatient register and 175 in the SCR. Some patients were identified in multiple registries; hence, the total sum is larger than the number of individuals in the final study population. Overall, the most common recorded subtype, in total 853 (42%), was UP/MPCM (ICD-10 code Q822A, *n* = 804 NPR outpatients and *n* = 49 NPR inpatients) (Supplementary, Table 2).

#### Outpatient NPR

The majority of the 1,912 patients identified in the outpatient NPR were registered with UP/MPCM (ICD-10 code Q822A, *n* = 804; 42%) and mastocytosis (ICD-10 code Q822, *n* = 447; 23%), followed by histiocyte and mast cell tumors of unknown nature (ICD-10 code D470, *n* = 296; 15%).

#### Inpatient NPR

A total of 330 patients were identified in the inpatient NPR of which a majority were registered with mastocytosis (ICD-10 code Q882, *n* = 108; 33%). Seventy-one patients (21%) had histiocyte or mast cell tumor of unknown nature (ICD-10 code D470) and 49 (15%) UP/MPCM (ICD-10 code Q822A).

#### Swedish Cancer Register

In the Swedish Cancer Register, 175 patients were identified where the majority were recorded with a diagnosis of indolent systemic mastocytosis (SNOMED code 94711, *n* = 101; 58%).

### Demographic characteristics

Most patients were Swedish born (80.4%), with about 9% being born outside Europe, a distribution similar to that in the comparison cohort. There were no significant differences in educational level, income, or household size between cases and comparators. However, compared to comparators, a higher proportion of cases were married (Table 3).

**Table 3:** Sociodemographic characteristics in individuals with a mastocytosis diagnosis and comparators age ≥20 years at index date.

	Cases		Comparators		*p*
	*n*	%	*n*	%	
**Educational level**					0.10
Low	361	17.7	1969	19.3	
Middle	906	44.4	4347	42.6	
High	758	37.2	3761	36.9	
Missing	15	0.7	116	1.1	
**Family size**					0.5
1	777	38.1	3980	39.0	
2+	1263	61.9	6209	60.9	
Missing	0	0.0	4	0.0	
Median, IQR	2	1–2	2	1–2	
**Region of birth**					0.7
Sweden	1641	80.4	8269	81.1	
Nordics, excluding Sweden	85	4.2	370	3.6	
Europe excluding Nordics	138	6.8	673	6.6	
Rest of the world	176	8.6	881	8.6	
**Marital status**					0.05
Married/partner	1026	50.3	4843	47.5	
Unmarried	537	26.3	2855	28.0	
Divorced	329	16.1	1606	15.8	
Widow/Widower	148	7.3	885	8.7	
Missing	0	0.0	4	0.0	

### Comorbidity burden

Most individuals with mastocytosis (79.6%) were categorized as having no comorbidity (CCI = 0) at time of diagnosis, 8.2% had CCI = 1 and 12.3% CCI ≥ 2. The corresponding estimates in comparators were 88.0%, 5.4% and 6.6%, respectively.

The most frequently registered concomitant medical condition at time of mastocytosis diagnosis was cancer (8.5%), followed by diabetes (3.7%). Cancer was also the most frequently recorded disease in the comparison cohort group (3.7%). There were significant differences between cases and comparators not only in the frequency of cancer (including metastatic carcinoma) but also regarding diabetes, chronic pulmonary disease, paraplegia and hemiplegia and renal disease (Table 4).

**Table 4:** CCI and comorbidities in individuals with a mastocytosis diagnosis and comparators age ≥ 20 years at index date.

	Cases		Comparators		OR	95% CI	*p*	Total	
	*n*	%	*n*	%				*n*	%
**Charlson Comorbidity Index (CCI)**									
0	1623	79.6	8,970	88.0	0.53	0.47–0.60	<0.001	10593	86.6
1	167	8.2	555	5.4	1.55	1.29–1.85	<0.001	722	5.9
2+	250	12.3	668	6.6	1.99	1.71–2.32	<0.001	918	7.5
**Morbidities within CCI**									
Myocardial infarction	34	1.7	146	1.4	1.17	0.80–1.70	0.483	180	1.5
Congestive heart failure	34	1.7	163	1.6	1.04	0.72–1.51	0.901	197	1.6
Peripheral vascular disease	22	1.1	67	0.7	1.64	1.02–2.67	0.057	89	0.7
Cerebrovascular disease	64	3.1	259	2.5	1.24	0.94–1.64	0.145	323	2.6
Dementia	6	0.3	46	0.5	0.65	0.28–1.53	0.418	52	0.4
Chronic pulmonary disease	69	3.4	186	1.8	1.88	1.42–2.49	<0.001	255	2.1
Connective tissue disease-rheumatic disease	12	0.6	74	0.7	0.81	0.44–1.49	0.593	86	0.7
Peptic ulcer disease	16	0.8	58	0.6	1.38	0.79–2.41	0.323	74	0.6
Mild liver disease	11	0.5	49	0.5	1.12	0.58–2.16	0.864	60	0.5
Diabetes without complications	76	3.7	226	2.2	1.71	1.31–2.22	<0.001	302	2.5
Diabetes with complications	12	0.6	42	0.4	1.43	0.75–2.72	0.361	54	0.4
Paraplegia and hemiplegia	20	1.0	27	0.3	3.73	2.09–6.66	<0.001	47	0.4
Renal disease	21	1.0	44	0.4	2.39	1.42–4.04	0.001	65	0.5
Cancer	173	8.5	379	3.7	2.40	2.00–2.89	<0.001	552	4.5
Moderate or severe liver disease	1	0.0	7	0.1	0.71	not estimated	not estimated	8	0.1
Metastatic carcinoma	22	1.1	39	0.4	2.83	1.68–4.80	<0.001	61	0.5
AIDS/HIV	3	0.1	1	0.0	15.01	not estimated	not estimated	4	0.0

When assessed by subtype, a CCI score of **≥** 2 was more common in patients with advanced (30.2%) and mixed subtypes (16.0%) compared to benign disease (7.1%). Cancer was the most frequently reported concomitant condition in all subtypes, but most common in advanced disease (23.9%). (Supplementary, Table 3). A separate analysis also showed a significant difference between benign cases (5.0%) and their matched comparators (2.9%) with regard to cancer (data not shown).

### Overall survival among all subjects and by mastocytosis subgroups

**Figure 1:**
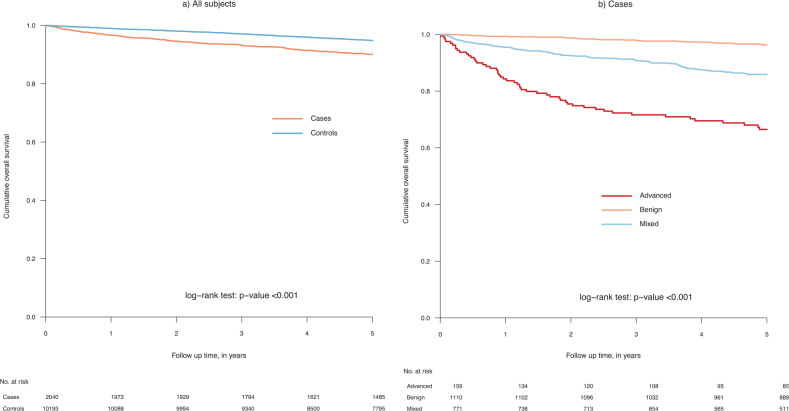
Overall survival in individuals with a mastocytosis diagnosis and comparators (controls) (A) and by mastocytosis subtype (B).

Compared to comparators the 5-year OS was lower in individuals with mastocytosis (90.1% vs. 94.8%; *p* < 0.001, HR 1.37). Five-year OS also differed between mastocytosis subtypes; advanced (66.5%), mixed (85.9%) and benign (96.3%) (*p* < 0.001) (Figure 1). Both patients with a record of an advanced and mixed subtype had a statistically significantly worse survival in comparison to their matched control group (supplementary, Figure 1).

### Cause of death

Among a total of 312 deaths, information on underlying cause of death was available for 285 mastocytosis patients. Of these, 87 patients had been registered with a diagnosis CM. The most common registered cause of death were malignant tumors (39%), excluding mast cell associated, and cardiovascular disease (26%). In only twelve patients, cause of death was registered as being related to mastocytosis, that is, mastocytosis, mast cell leukemia, malignant mast cell tumor and histiocyte and mast cell tumor of unknown nature. Of the three patients with mastocytosis (Q822) recorded as underlying cause of death, two were registered with a diagnosis of malignant mast cell tumor and one with mast cell tumor of unknown nature (Table 5).

**Table 5:** Cause of death in 285 individuals with a mastocytosis diagnosis.

ICD-10 code	*n* (%)
C-code, malignant tumors, excl mast cell associated	112 (39)
D-code, benign tumors, excl mast cells associated	21 (7.4)
I-code, cardiovascular diseases	74 (26)
Q822, mastocytosis	3 (1.0)
C943, mast cell leukemia	4 (1.4)
C962, malignant mast cell tumor	4 (1.4)
D470, histiocyte- and mast cell tumor of unknown nature	1 (0.0)
Others	66 (23)

## Discussion

Based on the hitherto largest population-based dataset, encompassing more than 2000 individuals aged 20 years or older, our results add to the understanding of the epidemiology of mastocytosis. In this original article, we found a clear female overrepresentation and a mean age at diagnosis of around 50 years. While mastocytosis is a condition that remains underdiagnosed, we found a higher incidence and prevalence compared to assessments in other geographic settings. We confirmed that the prognosis generally is favorable, but with marked survival differences between subtypes. Of special note was evidence of a higher comorbidity burden in mastocytosis patients compared to the background population. Except for marital status, there were no differences in background characteristics such as educational level, income, or country of birth between cases and individuals free of mastocytosis.

Improved knowledge, increased awareness, better use of diagnostic tools and the establishment of multidisciplinary centers are likely to have contributed to an increased detection and reporting of mastocytosis [20]. In this study, the prevalence and annual incidence for patients aged 20 years and older, was 23.9 and 1.56 per 100,000, respectively, estimates that exceeds results from earlier studies [8–12]. Possible explanations include the nationwide registry-based approach with data retrieved not only from the Cancer Register but also from the Patient Register based on diagnoses made both in in- and out-patient settings between 2001 and 2018. Centers of Excellence for the management of mastocytosis were established in Sweden in 2006 (Stockholm) and 2015 (Uppsala). In separate period estimates we found a higher incidence after the establishment of the first center.

Corroborating findings in earlier studies [3, 9], the highest survival was observed in patients with benign subtypes and an almost five times higher risk for death among advanced, compared to benign, subtypes. However, comparisons of survival between studies can be affected by differences in disease classifications used. We choose to include only SNOMED and ICD-codes clearly indicating advanced or benign disease. The codes for mastocytosis (Q822), mastocytosis unspecified (Q822X) and mast cell tumor of unknown nature (D470) were categorized as mixed and analyzed separately. Since the mixed group consisted of a large number of heterogeneous cases, they could have affected the survival estimates in the other categories, if they had been classified differently.

Similar to results in a nationwide Danish study [9], we found an overrepresentation of female patients. However, an Italian study reported a male predominance among patients managed in a reference center [11]. Similarly, a chart-based study from the Mayo clinic also showed a male overrepresentation [21]. One possible explanation is that men may be more readily referred to specialized centers.

The proportion of individuals with CCI = 0 was significantly higher in the comparison cohort. More than 12% of mastocytosis patients had CCI ≥ 2, compared to about 7% in comparators. Analysis by subtype, revealed that the proportion of patients with CCI ≥ 2 was higher in patients with advanced and mixed type SM compared to those with benign disease. Concomitant conditions at baseline more common in mastocytosis patients were cancer, including metastatic carcinoma, diabetes without complications, chronic pulmonary disease, renal disease and paraplegia and hemiplegia. However, there were no differences in rates of cardiovascular diseases, dementia, connective tissue disease, peptic ulcer and liver disease. Because of a variety of mast cell-derived symptoms and a high comorbidity burden in individuals with mastocytosis, a multidisciplinary approach [22] has been recommended with referral to specialized centers that can offer management tailored to the needs of each patient [23].

The major strengths of our study included the population-based setting and the size of the study population. Data were retrieved from several health registers with national coverage, based on diagnoses made in specialist care, minimizing the risk of incorrect assessments and selection bias. Also, mimicking differential diagnoses are few, minimizing the risk that the patients receive the diagnosis by mistake. The availability of randomly selected cohort free of mastocytosis provided an opportunity to make comparisons to the background population.

Several limitations need mentioning. While several Swedish nationwide register resources were combined to define the study population, not all individuals with mastocytosis were identified. Firstly, because of the rarity of the condition, signs and symptoms of mastocytosis remain unknown to some clinicians resulting in many cases being undetected [23]. Similar to other countries, some patients are seen only in primary care settings for which no data were available. Thus, like earlier studies, our estimates of incidence and prevalence are underestimated to an unknown extent. Secondly, the present study highlights the need of precise classification and categorization. When mastocytosis is diagnosed, there is a clinical challenge to correctly distinguish between subtypes, leading to possible misclassification of subtype. Many clinicians also appear to routinely use the ICD-10 codes for mastocytosis or mastocytosis unspecified. Since our study was based on ICD-10 and SNOMED codes retrieved from population-based registers, we were unable to validate the recorded subgrouping of mastocytosis by review of medical charts. While this limitation may have affected the subgroup survival estimates to an unknown extent, it should not affect the overall estimates for mastocytosis patients as a group. Thirdly, since the present study was conducted in a Swedish health care setting, the generalizability of our findings is unknown.

In conclusion, our results highlight the need of improved subclassification of mastocytosis and a better understanding of concomitant conditions in patients with a mastocytosis diagnosis. Of special interest is to explore not only common pathogenesis but also the possible influence of a higher detection rate of mastocytosis in individuals with certain conditions. In addition, the higher rates of cancer observed in mastocytosis patients compared to the background population warrant further studies.

## Supplementary Material

Epidemiology of mastocytosis: a population-based study (Sweden)

## Data Availability

Data cannot be shared for ethical/privacy reasons.

## References

[1] Valent P, Akin C, Hartmann K, et al. Updated diagnostic criteria and classification of mast cell disorders: a consensus proposal. HemaSphere. 2021;5:11. 10.1097/HS9.0000000000000646 PMC8659997 34901755

[2] Metcalfe DD. Mast cells and mastocytosis. Blood. 2008;112:946–56. 10.1182/blood-2007-11-078097 18684881 PMC2515131

[3] Valent P, Akin C, Metcalfe DD. Mastocytosis: 2016 updated WHO classification and novel emerging treatment concepts. Blood. 2017;129:1420–7. 10.1182/blood-2016-09-731893 28031180 PMC5356454

[4] Khoury JD, Solary E, Abla O, et al. The 5th edition of the World Health Organization Classification of haematolymphoid tumours: myeloid and histiocytic/dendritic neoplasms. Leukemia. 2022;36:1703–19. 10.1038/s41375-022-01613-1 35732831 PMC9252913

[5] Hartmann K, Escribano L, Grattan C, et al. Cutaneous manifestations in patients with mastocytosis: consensus report of the European Competence Network on Mastocytosis; the American Academy of Allergy, Asthma & Immunology; and the European Academy of Allergology and Clinical Immunology. J Allergy Clin Immunol. 2016;137:35–45. 10.1016/j.jaci.2015.08.034 26476479

[6] Valent P, Akin C, Escribano L, et al. Standards and standardization in mastocytosis: consensus statements on diagnostics, treatment recommendations and response criteria. Eur J Clin Invest. 2007;37:435–53. 10.1111/j.1365-2362.2007.01807.x 17537151

[7] Berezowska S, Flaig MJ, Ruëff F, et al. Adult-onset mastocytosis in the skin is highly suggestive of systemic mastocytosis. Mod Pathol Off J U S Can Acad Pathol Inc. 2014; 27: 19–29. 10.1038/modpathol.2013.117 23807778

[8] van Doormaal JJ, Arends S, Brunekreeft KL, van der Wal VB, et al. Prevalence of indolent systemic mastocytosis in a Dutch region. J Allergy Clin Immunol. 2013;131:1429–31. 10.1016/j.jaci.2012.10.015 23219169

[9] Cohen SS, Skovbo S, Vestergaard H, et al. Epidemiology of systemic mastocytosis in Denmark. Br J Haematol. 2014;166:521–8. 10.1111/bjh.12916 24761987

[10] Kibsgaard L, Deleuran M, Flohr C, Langan S, Braae Olesen A, Verstergaard C. How ‘benign’ is cutaneous mastocytosis? A Danish registry-based matched cohort study. Int J Womens Dermatol. 2020;6:294–300. 10.1016/j.ijwd.2020.05.013 33015290 PMC7522902

[11] Zanotti R, Bonifacio M, Isolan C, et al. A multidisciplinary diagnostic approach reveals a higher prevalence of indolent systemic mastocytosis: 15-years’ experience of the GISM network. Cancers. 2021;13:6380. 10.3390/cancers13246380 34944999 PMC8699786

[12] Ungerstedt J, Ljung C, Klimkowska M, Gülen T. Clinical outcomes of adults with systemic mastocytosis: a 15-year multidisciplinary experience. Cancers. 2022;14:3942. 10.3390/cancers14163942 36010937 PMC9405903

[13] Gülen T, Akin C. Anaphylaxis and mast cell disorders. Immunol Allergy Clin North Am. 2022;42:45–63. 10.1016/j.iac.2021.09.007 34823750

[14] Greene LW, Asadipooya K, Corradi PF, Akin C. Endocrine manifestations of systemic mastocytosis in bone. Rev Endocr Metab Disord. 2016;17:419–31. 10.1007/s11154-016-9362-3 27239674

[15] Hägglund H, Sander B, Gülen T, Lindelöf B, Nilsson G. Increased risk of malignant melanoma in patients with systemic mastocytosis? Acta Derm Venereol. 2014; 94: 583–584. 10.2340/00015555-1788 24473924

[16] Broesby-Olsen S, Farkas DK, Vestergaard H, et al. Risk of solid cancer, cardiovascular disease, anaphylaxis, osteoporosis and fractures in patients with systemic mastocytosis: a nationwide population-based study. Am J Hematol. 2016;91:1069–75. 10.1002/ajh.24490 27428296

[17] Indhirajanti S, van Daele PLA, Bos S, Mulder MT, Bot I, Roeters van Lennep JE. Systemic mastocytosis associates with cardiovascular events despite lower plasma lipid levels. Atherosclerosis. 2018;268:152–6. 10.1016/j.atherosclerosis.2017.11.030 29227868

[18] Charlson ME, Pompei P, Ales KL, MacKenzie CR. A new method of classifying prognostic comorbidity in longitudinal studies: development and validation. J Chronic Dis. 1987;40:373–83. 10.1016/0021-9681(87)90171-8 3558716

[19] Quan H, Li B, Couris CM, et al. Updating and validating the Charlson comorbidity index and score for risk adjustment in hospital discharge abstracts using data from 6 countries. Am J Epidemiol. 2011;173:676–82. 10.1093/aje/kwq433 21330339

[20] Valent P, Hartmann K, Bonadonna P, et al. European Competence Network on Mastocytosis (ECNM): 20-year Jubilee, updates, and future perspectives. J Allergy Clin Immunol Pract. 2023;11(6):1706–1717. 10.1016/j.jaip.2023.02.021 36868470

[21] Lim K-H, Tefferi A, Lasho TL, et al. Systemic mastocytosis in 342 consecutive adults: survival studies and prognostic factors. Blood. 2009; 113: 5727–36. 10.1182/blood-2009-02-205237 19363219

[22] Valent P, Akin C, Gleixner KV, et al. Multidisciplinary challenges in mastocytosis and how to address with personalized medicine approaches. Int J Mol Sci. 2019;20(12):2976. 10.3390/ijms20122976 31216696 PMC6627900

[23] Broesby-Olsen S, Dybedal I, Gülen T, et al. Multidisciplinary management of mastocytosis: Nordic Expert Group consensus. Acta Derm Venereol. 2016;96:602–12. 10.2340/00015555-2325 26694951

