# Supplementary material for: Epidemiology of mastocytosis: a population-based study (Sweden)
Source: Acta Oncol. 2024 Feb 21;63:31406. doi: 10.2340/1651-226X.2024.31406 (PMC11332469; doi:10.2340/1651-226X.2024.31406)

Supplementary material has been published as submitted. It has not been copyedited or typeset by Acta Oncologica.

Supplementary

Supporting information appendix

ICD and SNOMED codes used to identify cohort of mastocytosis patients

Diagnosis	ICD-9	ICD-10	SNOMED
Cutaneous mastocytosis		Q822	97401
- Urticaria pigmentosa		Q822A	
- DCM		Q822B	
- Mastocytoma	238F	Q822C	
- TMEP		Q822D	
- Mastocytosis unspec		Q822X	
Systemic mastocytosis		Q822	97411, 97413
Malignant mastocytosis	202G		
Mast cell leukemia		C943	97423
Malignant mast cell tumor, Aggressive SM, SMAHN		C962	97413
Mast cell tumor of unknown nature, ISM		D470	97411
Mast cell sarcoma		C962	97403

Registries used for our dataset

Register	Data content
Swedish Cancer Register	Notification of cancer diagnosis, site and date, according to SNOMED.

Patient Register	In- and outpatient Registers, diagnosis according to ICD, revision 7-10.
Cause of Death Register	Date and underlying/contributing causes of death, according to ICD-codes.
Register of Total Population	Data of the composition, family relationships and identities of the Swedish population.
Longitudinal database on socio-economic factors (LISA)	Socioeconomic factors, including income, civil status, type of employment and income

Supplementary tables

Table 1. Demographics, all subjects between index year of 2001-2018

	All subjects	
**All subjects, n /%**	4438	100
**Data sources**	n	%
**NPR, outpatient care, ICD-codes**	4292	% of all subjects in NPR outpatient
C943	13	0.3
C962	96	2.2
D470	405	9.4
Q822	1059	24.6
Q822A	1313	30.5
Q822B	57	1.3
Q822C	977	22.7
Q822D	99	2.3
Q822X	273	6.3
**NPR, Inpatient care, ICD-codes**	390	% of all subjects in NPR inpatient
238F	1	0.0
C943	6	0.7
C962	42	10.7
D470	79	20.2
Q822	137	35.1
Q822A	59	15.1
Q822B	32	8.2
Q822C	4	0.1
Q822D	3	0.1
Q822X	27	6.9
**Cancer registry, SNOMED-codes**	186	% of all subjects in SCR
97401	26	13.9
97403	2	1.0

97411	105	56.4
97413	50	26.8
97423	3	1.6
**Sex**		
Male	2245	50.6
Female	2193	49.4
**Age at index date**		
Median/IQR	12	1-47
Mean/sd	25	26.6
**Age group**	n	%
0-1 (infants)	1237	27.9
2-9	923	20.8
10-19	238	5.4
20-29	284	6.4
30-39	328	7.4
40-49	388	8.7
50-59	365	8.2
60-69	345	7.8
70-79	227	5.1
80-89	91	2.1
90+	12	0.3

Table 2. Distribution of subtypes by register source in individuals (≥20 years) with a mastocytosis diagnosis

**Data source**	n	%
**NPR, outpatient care, ICD-codes**	1912	% of all subjects in NPR outpatient
C943	9	0.5
C962	61	3.1
D470	296	15.4
Q822	447	23.3
Q822A	804	42.0
Q822B	28	1.5
Q822C	56	2.9
Q822D	88	4.6
Q822X	123	6.4
**NPR, Inpatient care, ICD-codes**	330	% of all subjects in NPR inpatient
238F	1	0.0
C943	3	0.1
C962	39	11.8
D470	71	21.5
Q822	108	32.7
Q822A	49	14.8
Q822B	31	9.3
Q822C	1	0.0
Q822D	3	0.1
Q822X	24	7.3
**Cancer Register, SNOMED codes**	175	% of all subjects in SCR
97401	19	10.8
97403	2	1.1
97411	101	57.7
97413	50	28.6
97423	3	1.7

Table 3. CCI and comorbidities by subtype

	Advanced<br>n=159	Benign<br>n=1110	Mixed<br>n=771	p-value
**Charlson Comorbidity Index (CCI)**				<0.001
0	101 (63.5)	960 (86.5)	562 (72.9)	
1	10 (6.3)	71 (6.4)	86 (11.2)	
2+	48 (30.2)	79 (7.1)	123 (16.0)	
**Morbidities within CCI**				
Myocardial Infarction	4 (2.5)	14 (1.3)	16 (2.1)	0.273
Congestive Heart Failure	3 (1.9)	12 (1.1)	19 (2.5)	0.068
Peripheral Vascular Disease	3 (1.9)	6 (0.5)	13 (1.7)	0.036
Cerebrovascular Disease	2 (1.3)	20 (1.8)	42 (5.4)	<0.001
Dementia	0 (0.0)	2 (0.2)	4 (0.5)	0.319
Chronic Pulmonary Disease	5 (3.1)	25 (2.3)	39 (5.1)	0.004
Connective Tissue Disease-Rheumatic Disease	2 (1.3)	5 (0.5)	5 (0.6)	0.443
Peptic Ulcer Disease	1 (0.6)	6 (0.5)	9 (1.2)	0.309
Mild Liver Disease	0 (0.0)	6 (0.5)	5 (0.6)	0.596
Diabetes without complications	6 (3.8)	31 (2.8)	39 (5.1)	0.039
Diabetes with complications	1 (0.6)	3 (0.3)	8 (1.0)	0.101
Paraplegia and Hemiplegia	0 (0.0)	3 (0.3)	17 (2.2)	<0.001
Renal Disease	4 (2.5)	3 (0.3)	14 (1.8)	0.001
Cancer	38 (23.9)	55 (5.0)	80 (10.4)	<0.001
Moderate or Severe Liver Disease	0 (0.0)	0 (0.0)	1 (0.1)	0.439
Metastatic Carcinoma	9 (5.7)	5 (0.5)	8 (1.0)	<0.001
AIDS/HIV	0 (0.0)	3 (0.3)	0 (0.0)	0.284

Figure 1. Overall survival by mastocytosis subtype with their matched comparators (controls).

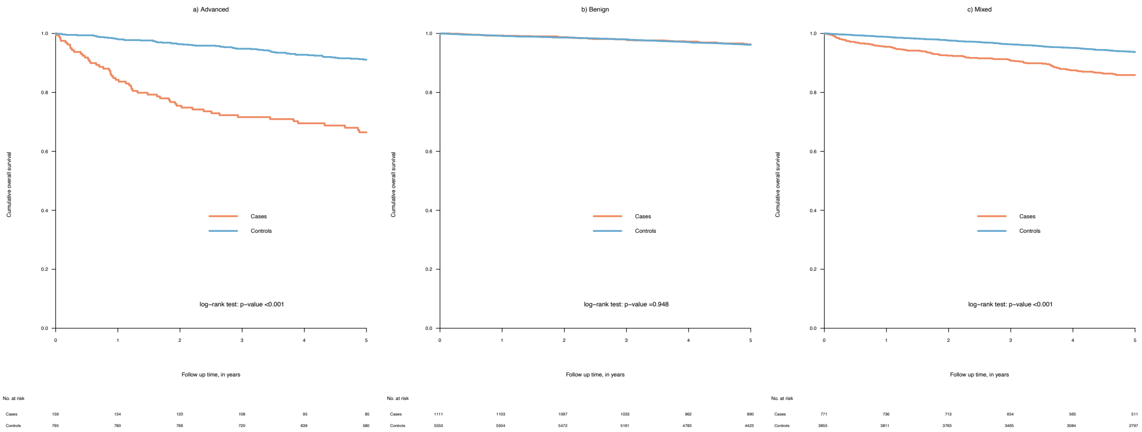

Supplement: Epidemiology of mastocytosis: a population-based study (Sweden) [file AO-63-31406-s1.pdf]
